# Optimizing assessors’ mental workload in rater-based assessment: a critical narrative review

**DOI:** 10.1007/s40037-019-00535-6

**Published:** 2019-11-14

**Authors:** Bridget Paravattil, Kyle John Wilby

**Affiliations:** 1 College of Pharmacy, Qatar University, Doha, Qatar; 2 School of Pharmacy, University of Otago, Dunedin, New Zealand

**Keywords:** Mental workload, Cognitive load, Assessment

## Abstract

**Introduction:**

Rater-based assessment has resulted in high cognitive demands for assessors within the education of health professionals. Rating quality may be influenced by the mental workload required of assessors to complete rating tasks. The objective of this review was to explore interventions or strategies aimed at measuring and reducing mental workload for improvement in assessment outcomes in health professions education.

**Methods:**

A critical narrative review was conducted for English-language articles using the databases PubMed, EMBASE, and Google Scholar from conception until November 2018. To be included, articles were eligible if they reported results of interventions aimed at measuring or reducing mental workload in rater-based assessment.

**Results:**

A total of six articles were included in the review. All studies were conducted in simulation settings (OSCEs or videotaped interactions). Of the four studies that measured mental workload, none found any reduction in mental workload as demonstrated by objective secondary task performance after interventions of assessor training or reductions in competency dimension assessment. Reductions in competency dimensions, however, did result in improvements in assessment quality across three studies.

**Discussion:**

The concept of mental workload in assessment in medical education needs further exploration, including investigation into valid measures of assessors’ mental workload. It appears that adjusting raters’ focus may be a valid strategy to improve assessment outcomes. Future research should be designed to inform how to best reduce load in assessments to improve quality, while balancing the type and quantity of data needed for judgments.

## What this paper adds

Mental workload in rater-based assessment is speculated to negatively influence rating quality yet the implications of intervening to reduce mental workload are unknown. The literature to date provides differing theoretical perspectives (Cognitive Load Theory, Information Processing Theory) regarding the influence of mental workload in assessment but empirical data are lacking. This critical narrative review bridges theory to practice by identifying, synthesizing, and evaluating the available empirical evidence that reports interventions targeted to reducing mental workload in rater-based assessment.

## Introduction

The era of competency-based health education has resulted in high assessment burdens [1, 2]. The advent of new assessment contexts, such as the objective structured clinical examination (OSCE), in-training evaluation of clinical skills, and now entrustable professional activities (EPAs) place high demands on students but are also demanding of the assessors who must observe, synthesize, and ultimately judge performance [3, 4]. Assessors are being asked to complete checklists, rubrics, rating scales, and write comments relating to the tasks, competencies, or activities being assessed. At times, these assessment procedures can encompass multiple tasks, competencies, or activities within one observation occurrence or require assessors to disentangle student performance across these tasks, competencies, or activities after extended periods of clinical observation [5, 6]. This results in high cognitive demands from the assessor, which can be further compounded by complexity embedded within assessment tools or forms [7].

The problem of assessment burden in the education of health professionals is well documented, yet solutions seem to be few and far between [3]. Increasing demands from accrediting and professional bodies for programs to provide evidence that graduates are achieving intended competencies or program learning outcomes is resulting in a heavy reliance on rater-based assessment, especially within the context of clinical training [8, 9]. The high burden imposed by competency-based assessment, especially in a summative context, may increase assessors’ reliance on memory and impair their ability to provide accurate and meaningful feedback for student performance [10, 11]. Despite widespread recognition of this problem across health professions education, no clear answer exists regarding the best approach to reduce the burden of assessment and improve the quality of ratings we receive from our assessors [3].

Throughout the past six years, there has been an increase in the amount of literature attempting to better understand assessor burden and to explore the effects of interventions aimed to improve assessment quality. The theoretical underpinnings that appear to be driving this research relate to assessors’ mental workload, the cognitive load experienced by assessors during an assessment task, and the cognitive processes required to complete the assessment task [5, 12, 13]. Tavares and Eva originally proposed that relating perceptual load theory, cognitive load theory, and information processing theory to clinical performance ratings may help to improve rating quality [5]. More specifically, these authors suggest that the effort, or load, required for rating tasks should be evaluated and aligned with cognitive capacity. It is proposed that reducing competing demands and/or reducing complexity of the rating task may result in higher quality ratings. Others have proposed differing perspectives. Wood (2013) argues that it may be less about finding the ‘bottleneck’ in terms of the cognitive demand or complexity but more about the cognitive *processes* that are required for raters to make judgments and that modifying the rating tasks may influence how raters actually perform what they are required to do [12]. Despite differences in these perspectives, a common thread is that there seems to be agreement that mental workload may influence rating tasks.

Given the importance of mental workload in rater-based assessments, targeted interventions to reduce workload may improve rating quality. According to cognitive load theory, there may be three different types of mental workload to consider: intrinsic, extrinsic, and germane. Intrinsic load has been described as the complexity of the task, which is measured by the extent of association between the learner’s expertise and the nature of the task. From the context of an assessor, examples of intrinsic load may include recalling the scenario or evaluating various competencies at one time [5]. Extraneous load is defined as the load that is imposed due to poor instructional design or other factors that divert the attention away from the learning environment. Assessors may be exposed to this type of load when given an assessment tool that is not clear or when asked to perform secondary assessment tasks, such as assessing the performance of a simulated actor or being responsible for operating timers or audiovisual aids during an assessment [5]. Germane load has been explained as the cognitive effort necessary for learning, which differs from the interference nature of both intrinsic and extrinsic load. Germane load intentionally imposes a cognitive effort that changes the nature of the task to enhance the learning process. In assessment, this simply means the mental effort that the assessor dedicates to the rating task [5]. Each of these types of load may be the target of interventions to reduce mental workload and improve assessment quality.

Given the amount of empirical data now published in medical education relating to mental workload and assessment, the objective of this critical narrative review was to summarize the findings of existing empirical research on assessors’ cognitive load within the health professions.

## Methods

This was a critical narrative review of published literature in health professions education. A critical narrative review was conducted due to the nature of the objective (i.e. focus on empirical studies), and the diversity of study designs known to address this phenomenon [14]. A search of PubMed, EMBASE, and Google Scholar without date limits until March 2019 was conducted using the search terms mental workload, cognitive load, mental capacity, evaluation fatigue, rater fatigue, and assessment. Search terms were extracted from key studies known to the authors in the field of mental workload and assessment. Search terms were combined with OR, aside from assessment, which was combined with all other search terms using AND. All search terms were limited to Title/Abstract. Search results were limited to those published in English. The electronic search was supplemented with a manual search of the reference lists from identified relevant studies and/or review articles. Articles were screened for eligibility independently by two investigators. Articles were eligible if they reported results of interventions aimed at measuring mental workload in health professions education assessment or interventions aimed at reducing mental workload associated with assessment tasks in health professions education. Conference abstracts were excluded. Discrepancies between investigators inclusion of an article were resolved by discussion. Data were extracted from each included article using an extraction spreadsheet. Extraction points included author, title, year of publication, aims, population, context/setting, interventions, outcomes, and results. One investigator (BP) extracted all data and the second investigator verified the data (KW). Both investigators met via videoconference on multiple occasions over a 3-month period to discuss articles and interpret findings.

## Results

**Fig. 1:**
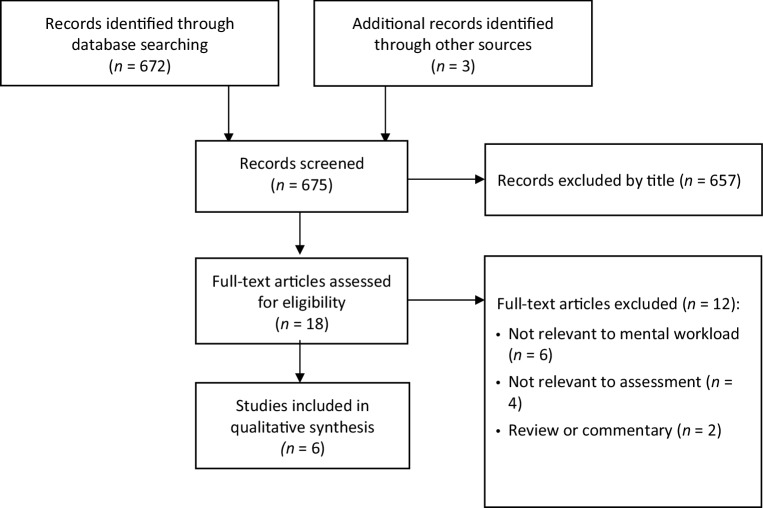
Flow chart of included studies

Electronic database searching resulted in 672 hits, of which 18 were identified through title and abstract screening for full-text review. An additional 3 articles were identified from manual searching of reference lists. After full-text review, we identified 6 studies that reported empirical interventions aimed to reducing mental workload within the context of medical education and met our inclusion criteria [15–20]. The search strategy is outlined in Fig. 1.

A key concept that must be identified before reviewing the studies is that of measurement of mental workload. The studies identified used two different approaches. First, workload was measured subjectively using the NASA Task Load Index (TLX) instrument. The NASA-TLX subjective workload questionnaire is a subjective, self-reported, multidimensional assessment that rates a participant’s perceived workload on a given task [21]. Secondly, workload was measured using performance data from an objective secondary task [5, 21]. This task was meant to be an objective measure of a surrogate endpoint presumed to enable conclusions to be drawn about mental workload. A wireless vibrotactile device was placed on the arm of each participant. The device then vibrated at random intervals between 10 and 90 s over the course of the primary task participants were performing. Subjects needed to press the button on the device to cease it from vibrating whenever the device alerted but were also instructed to prioritize their primary task first. The time it took the participant to turn off the vibration was measured as a marker of the workload of the primary task (e.g. greater amounts of time meaning greater workload on the primary task).

Four of the six studies focused on measuring mental workload as a primary study outcome [15–18]. Byrne et al. (2014) attempted to measure mental load during a four-station OSCE where assessors were tasked with rating student performance using 21–22 item checklist [15]. Mental load was evaluated by the examiners’ response time to the stimulus produced by the vibrotactile device, which was strapped to the assessor’s arm and set to vibrate every 10–90 s during the assessment. Scores obtained from the NASA-TLX were also used to measure assessors’ mental load. A comparator group of 24 trainee anaesthetists was selected as their tasks were deemed to be similar to those required of an OSCE examiner and of high mental workload. Findings showed longer delays in vibrotactile response times and higher median NASA-TLX among the examiners, as compared with the trainee anaesthetists. Based on these findings, the authors concluded that the mental workload of examiners is excessive in the OSCE setting. Although this study had a small sample size and dissimilar study conditions between the groups, it set the stage for further research to determine acceptable levels of mental workload and whether assessor training or redesigning of assessments would be effective alternatives.

Byrne et al. (2016) completed a second study, which aimed to determine the impact of assessor training on reduction of mental workload and improvement of assessor accuracy [16]. Assessors were selected from a training program that required them to rate four different video scenarios according to an assessment checklist. After each video, assessors compared marks with other trainees and received feedback from an experienced examiner. Accuracy of ratings was defined as the difference between the ratings of the participant and those of an expert group, which had previously reached consensus on ‘true scores’. Mental workload was measured using the vibrotactile device, as described above. The study found that training did not improve the accuracy of ratings and that there was also no effect on mental workload, as measured by the vibrotactile device. The authors attribute these findings to the high mental workload nature of the OSCE and speculate that a single training session does not result in sufficient training to promote improved accuracy in scores.

Tavares et al. (2014) completed a study to evaluate how increasing rating demands impacts rater-based assessment of clinical competence using pre-recorded clinical encounters [17]. Participants were randomized to one of four conditions: two or seven rating dimensions with the presence or absence of extraneous tasks as distractors. Outcomes included the number of dimension-relevant behaviours identified, performance discrimination ability, and inter-rater reliability. Mental workload was measured using the vibrotactile device as well as the NASA-TLX. Findings showed no association between measurement of mental workload and each of the procedural conditions. However, more behaviours were identified in the groups rating performance across two dimensions as compared with seven dimensions. It was also found that those in the two-dimension groups had a better performance discrimination and inter-rater reliability versus those asked to rate across seven dimensions. The presence or absence of extraneous distracting tasks had no influence on measured outcomes. The study had limitations, however, due to the use of senior students as raters, as these novice raters may have different knowledge and understanding of the competency domains used for assessment. This limitation led to an additional study by Tavares and colleagues that recruited expert raters for competency assessment.

In the extension to the previous study, Tavares et al. (2016) explored how increasing task demands would influence rating quality among experienced raters [18]. Experienced assessors were randomized to rate pre-recorded videos of clinical encounters across two or seven dimensions with or without a task to increase extraneous load by requiring assessors to also rate the performance of the standardized actors. Findings were similar to the previous study, however, with greater inter-rater reliability being attributed to those rating across only two dimensions. The requirement of an additional rating task did not have any effect on rating quality but participants noted they blocked out this task, likely in an attempt to reduce extraneous load. The authors also interviewed participants to better understand how raters manage the workload imposed on them. Results from this analysis showed that raters use multiple strategies to reduce load to navigate the rating tasks, including prioritization, simplification and minimization of their perceived extraneous load. Despite, once again, no measured reduction in mental workload in this study, findings demonstrated improved assessment quality when the rating task was simplified and that assessors’ perceived ways of reducing their own mental workload may indeed be related to cognitive load and information processing theories.

Two additional studies were identified that further looked at the practical applications of reducing load within an assessment context [19, 20]. Hurley et al. (2015) completed a study to investigate checklist length on rater accuracy when assessing student performance on OSCEs [19]. Participants were recruited to watch four videos enacting 10-min integrated history and physical exam OSCE stations and were randomized to assess the videos according to either a 20- or 40-item checklist, which consisted of binary outcomes. Findings showed no difference in accuracy or inter-rater reliability between the 20- and 40-item checklist, suggesting simplifying a checklist by length alone may not be a viable strategy for reducing assessment load. It should be noted, however, that overall mean rater accuracy was 86% and therefore any minor (yet important) variations in scoring may not have been detected.

Tavares et al. (2018) expanded on previously reported studies with a study designed to determine if collecting sequential ratings across subsets of a candidate’s competencies, as opposed to having raters evaluate a greater number of competencies simultaneously, altered feedback and performance ratings [20]. Participant assessors were randomized to a sequential (rating across two dimensions) group or a simultaneous (rating across six dimensions) group for evaluation of video performances in a 3:1 fashion. Randomization was designed to ensure that three different assessors rated students in the sequential group (i.e. one for each pair of dimensions), in order to provide a complete evaluation over the six competencies. Participants were instructed to rate four clinical performances and to provide the feedback that they would give to the students in each video. Outcomes were the amount and type of feedback and the reliability of the scores. It was found that assessors in the sequential group provided greater amounts of feedback, greater variety in feedback, and there was greater breadth to the feedback across all competencies. Score reliability was also greater in the sequential group. Findings therefore suggested that simplification of assessment procedures once again resulted in positive outcomes, including improved feedback for trainees and/or clinical competency decision-makers.

## Discussion

The purpose of this review was to explore the interventions used to measure or reduce mental workload within assessments. Six studies were identified that provided an overview of published empirical research in this area [15–20]. Despite no clear link between task simplification and measurement of mental workload the studies identified show us how simplification of the assessment process may improve other assessment outcomes, such as interrater reliability and the quantity and quality of provided feedback. While many questions still remain, these studies provide a foundation for the design of future studies aimed to improve assessment quality by using different approaches to reduce the mental workload associated with assessment tasks.

The studies summarized above have both important theoretical and practical implications. From a theoretical perspective, the studies provide conflicting evidence for the explanation of mental workload as a limitation that impairs assessor performance. Based on the findings from studies that attempted to measure mental workload, it would appear that improvements in assessment quality markers are not associated with reductions in reported levels of mental workload [16–18]. That being said, it could also be argued that the ways in which mental workload is measured (vibrotactile device and NASA-TLX) are either not valid measures of mental workload or may not be sensitive enough to measure changes in mental workload observed within the studies. This argument aligns with Naismith et al. (2015) who showed validity evidence for measuring mental workload (self-reports, secondary task performance, physiological indices, and observer ratings) from learner perspectives in simulation settings was low [22]. It should be noted, however, that the studies reviewed were conducted outside the realm of assessment. It could also be speculated that the small sample sizes of the studies identified did not provide enough power to detect changes in outcomes.

The lack of a difference pertaining to assessor training found by Byrne et al. (2016) does not allow us to conclude more widely about the potential relationship between training and mental workload [16]. Despite limited data showing assessor training can improve assessment quality [3, 23], perhaps further training and/or experience may be necessary before mental workload is reduced and can be measured. Two studies by Tavares and colleagues also resulted in surprisingly absent findings when assessors were instructed to complete secondary tasks [17, 18]. The presence of the secondary tasks could have been expected to alter assessment quality due to their distractive nature, but little effect was noted. On the other hand, the studies by Tavares and colleagues do signal that mental workload may be a possible explanation for reduced assessment quality, as the studies consistently demonstrated that simplification of tasks results in better quality markers [17, 18, 20]. This may align with research from other disciplines that suggests load may be important for deliberate tasks, rather than those tasks that are more basic or typical [24].

If we take a closer look at assessor cognition literature, there could be other explanations that underpin the paradox found in this review. It is possible, for example, that assessor expertise, rather than attention and/or working memory as part of mental workload, may be more influential towards the quality of rating tasks [10, 25]. Although these concepts may be inter-related, expertise is known to encompass assessor characteristics, assessor perceptions of the assessment tasks, and the assessment context, rather than traditional components of mental workload (attention, working memory, information processing). Increasing literature is also showing that assessor beliefs, performance theories, and inferences about the student may be contributing to their reasoning of student performance and the assessment task [26, 27]. If the outcome of assessors’ ratings is largely shaped by these factors, rather than what they pay attention to or remember from a performance, it could provide an explanation as to why deliberate manipulations to reduce mental workload appear to have little impact on assessment quality, as largely measured by inter-rater reliability. As Schutz and Moss (2004) argue, variability between assessors appears to be less about what they focus on or pay attention to and more about how they conceptualize and bring meaning to data by developing a coherent representation or story about student performance [28]. If this is indeed true, perhaps the process in which assessors interpret and bring meaning to performance data is not dependent on reductions in mental workload. It could then be argued that by simplifying rating tasks by reducing the number of competencies an assessor is asked to consider, assessors may become more congruent in their processing of performance information, leading to the positive outcomes observed in the studies by Tavares et al. [17, 18, 20]. Although there could be other possible theoretical explanations for these findings, such as pushing assessors ‘outside their comfort zone’ in how they normally perceive competence when making judgments [29], the concept of mental workload and its influence on rating demands is likely to be at least contributory to our understanding of the burdens placed on assessors through rater-based assessment and warrants further investigation.

From a practical perspective, the main message arising from this review is that simplification of rater-based assessments may be the key to improving assessment quality and capturing a better perspective of trainee performance through both rating and provision of feedback. The studies by Tavares et al. have shown that reducing assessors’ focus based on competency dimension allows for better assessment markers [17, 18, 20]. This finding is in contrast to Hurley et al. where the intervention was to reduce the number of checklist items, rather than the number of competencies [19]. The key difference of these two findings is that in the checklist approach, assessors were still required to observe performance and provide judgment across the entire performance. The reductions in checklist items were based on deemed importance, rather than targeting specific performance dimensions. Tavares and colleagues instead attempted to focus the assessor’s attention on specific competency domains, which may have provided a better ‘signal’ for assessment and less ‘noise’ that may have interfered with judgments for other competency dimensions. It is still unclear, however, if other simplification strategies could be viable alternatives to Tavares’ approach and thus this should be a priority for future research.

Despite these positive findings, many questions remain regarding the practicality and feasibility of assessment simplification in practice. First, all studies to date have been based on video-captured assessments and research is yet to move into real-life practice. As such, there may be other factors that could affect the reproducibility of these results in a practice-based setting. Perhaps most importantly, social factors and relationship dynamics will be present in clinical training encounters between raters and trainees that are not accounted for in simulated interactions [30]. Simply asking assessors to only focus on a few competency dimensions after observing trainees over an extended period of time may be difficult for them to disentangle what they have also observed across the different competency dimensions, especially if assessors are not being asked to rate patient care performance. A second question to be explored is the feasibility of sequential assessment, as outlined in the final study by Tavares et al. [20]. The practicality of having assessors rate only a subset of competency dimension may be difficult for a) determining the number of times a student should be rated on a single competency dimension and b) ensuring the student is performing to the best of their abilities at all times (i.e. patient care) and not just according to when each competency is assessed. Despite these questions, the findings of this review support further research in this area to refine assessment practices and explore a more simplified approach to reduce rater demands.

This review should be interpreted in light of some limitations. First, our aim was to explore the association between mental workload and assessment, which resulted in identification of studies that discussed or used these terms as keywords. We therefore may have missed some studies looking at simplification of checklists or other assessment tools. Secondly, our review is limited by the quality of studies identified. In particular, measurement of mental workload was limited to the use of the vibrotactile device and NASA-TLX scoring tool. Despite previous validation of these methods, they may not have been sensitive enough to detect changes in mental workload demonstrated within the small sample sizes of the studies included in this review or as discussed, not be valid measures of workload associated with assessment in the health professions. Thirdly, our search strategy did not include specific assessment format terms (e.g. OSCE) but based on our findings, we are confident we identified most (if not all) studies that met our inclusion criteria. Lastly, research in this area is still expanding but with what appears to be a movement away from measuring mental workload; we believe our results provide a comprehensive summary and foundation for new research questions arising to address rater demands in assessment design.

The burden of assessment tasks will continue to increase as new models and methods are developed to train and assess students in both simulated and experiential settings. This review found that interventions aimed to reduce mental workload associated with these assessment tasks can improve assessment quality but the mediating role of load remains yet to be demonstrated within a medical education context. Moving forward, the concept of mental workload therefore remains theoretically important and more research is needed to better understand the relationship between mental workload and assessment quality. Specifically, studies should be designed to inform how to best reduce load in assessment to improve assessment quality, while balancing the type and amount of data needed for judgments. There is also an urgent need for research to move into the workplace-based setting, as many context-specific factors must be considered to ensure feasibility of research findings to date.
